# Quantitation of TIMP-1 in plasma of healthy blood donors and patients with advanced cancer

**DOI:** 10.1038/sj.bjc.6690384

**Published:** 1999-05-01

**Authors:** M N Holten-Andersen, G Murphy, H J Nielsen, A N Pedersen, I J Christensen, G Høyer-Hansen, N Brünner, R W Stephens

**Affiliations:** The Finsen Laboratory, Copenhagen University Hospital, Strandboulevarden 49, 2100 Copenhagen, Denmark; Strangeways Laboratories, Cambridge University, Cambridge, UK; Department of Oncology, Herlev Hospital, Herlev Ringvej, 2730, Herlev, Denmark; Department of Gastroenterology, Hvidovre Hospital, Kettegårds Alle, 2650 Hvidovre, Denmark

**Keywords:** TIMP-1, ELISA, plasma, cancer, blood donors

## Abstract

A kinetic enzyme-linked immunosorbent assay (ELISA) for plasma tissue inhibitor of metalloproteinase (TIMP)-1 was developed in order to examine the potential diagnostic and prognostic value of TIMP-1 measurements in cancer patients. The ELISA enabled specific detection of total TIMP-1 in EDTA, citrate and heparin plasma. The assay was rigorously tested and requirements of sensitivity, specificity, stability and good recovery were fulfilled. TIMP-1 levels measured in citrate plasma (mean 69.2 ± 13.1 μg l^−1^) correlated with TIMP-1 measured in EDTA plasma (mean 73.5 ± 14.2 μg l^−1^) from the same individuals in a set of 100 healthy blood donors (Spearman's rho = 0.62, *P* < 0.0001). The mean level of TIMP-1 in EDTA plasma from 143 patients with Dukes' stage D colorectal cancer was 240 ± 145 μg l^−1^ and a Mann–Whitney test demonstrated a highly significant difference between TIMP-1 levels in healthy blood donors and colorectal cancer patients (*P* < 0.0001). Similar findings were obtained for 19 patients with advanced breast cancer (mean 292 ± 331 μg l^−1^). The results show that TIMP-1 is readily measured in plasma samples by ELISA and that increased levels of TIMP-1 are found in patients with advanced cancer. It is proposed that plasma measurements of TIMP-1 may have value in the management of cancer patients. © 1999 Cancer Research Campaign


*British Journal of Cancer* (1999) **80**(3/4), 495–503
© 1999 Cancer Research Campaign
Article no. bjoc.1998.0384

### Quantitation of TIMP-1 in plasma of healthy blood donors and patients with advanced cancer

**MN Holten-Andersen[1], G Murphy[2], HJ Nielsen[4], AN Pedersen[1,3], IJ Christensen[1], G Høyer-Hansen[1], N Brünner[1] and RW Stephens[1]**

[1]The Finsen Laboratory, Copenhagen University Hospital, Strandboulevarden 49, 2100 Copenhagen, Denmark; [2]Strangeways Laboratories, Cambridge University, Cambridge, UK; [3]Department of Oncology, Herlev Hospital, Herlev Ringvej, 2730 Herlev, Denmark; [4]Department of Gastroenterology, Hvidovre Hospital, Kettegårds Alle, 2650 Hvidovre, Denmark

**Summary** A kinetic enzyme-linked immunosorbent assay (ELISA) for plasma tissue inhibitor of metalloproteinase (TIMP)-1 was developed in order to examine the potential diagnostic and prognostic value of TIMP-1 measurements in cancer patients. The ELISA enabled specific detection of total TIMP-1 in EDTA, citrate and heparin plasma. The assay was rigorously tested and requirements of sensitivity, specificity, stability and good recovery were fulfilled. TIMP-1 levels measured in citrate plasma (mean 69.2 ± 13.1 μg $l^{-1}$) correlated with TIMP-1 measured in EDTA plasma (mean 73.5 ± 14.2 μg $l^{-1}$) from the same individuals in a set of 100 healthy blood donors (Spearman's rho = 0.62, $P < 0.0001$). The mean level of TIMP-1 in EDTA plasma from 143 patients with Dukes' stage D colorectal cancer was 240 ± 145 μg $l^{-1}$ and a Mann–Whitney test demonstrated a highly significant difference between TIMP-1 levels in healthy blood donors and colorectal cancer patients ($P < 0.0001$). Similar findings were obtained for 19 patients with advanced breast cancer (mean 292 ± 331 μg $l^{-1}$). The results show that TIMP-1 is readily measured in plasma samples by ELISA and that increased levels of TIMP-1 are found in patients with advanced cancer. It is proposed that plasma measurements of TIMP-1 may have value in the management of cancer patients.

**Keywords:** TIMP-1; ELISA; plasma; cancer; blood donors

Matrix metalloproteinases (MMPs) play a pivotal role in cancer growth and spread, contributing to enzymatic degradation of the integrity of the extracellular matrix (Liotta et al, 1991; Stetler-Stevenson et al, 1993; MacDougall and Matrisian, 1995). The naturally occurring inhibitors of MMPs – tissue inhibitors of MMPs (TIMPs) form tight 1:1 stoichiometric complexes with the activated forms of the MMPs (Welgus et al, 1985; Kleiner et al, 1993) thereby inhibiting the catalytic activity of these enzymes (Goldberg et al, 1992; Birkedal-Hansen et al, 1993; Stetler-Stevenson et al, 1996). In support of a protective role of TIMP-1 and TIMP-2 it was found that in vitro invasiveness (Khokha et al, 1992a; Khokha and Waterhouse, 1993) and metastatic spread of tumour cells in experimental animal models (DeClerck et al, 1992; Khokha et al, 1992*b*) was inhibited by transfection of cancer cells with the genes encoding either TIMP-1 or TIMP-2. While the balance between the matrix-degrading properties of MMPs and the inhibitory effect of TIMPs is closely regulated under normal physiological conditions (Matrisian, 1992; Birkedal-Hansen et al, 1993; Thorgeirsson et al, 1993), this balance might be disrupted in malignant tissue.

A number of enzyme-linked immunoassays (ELISAs) for detection of TIMP-1 (Kodama et al, 1989; Cooksley et al, 1990; Clark et al, 1991) and TIMP-2 (Fujimoto et al, 1993) have been reported. These assays have been applied to various forms of bodily fluids, e.g. serum, plasma, amniotic fluid, cerebrospinal fluid and urine, but the number of samples tested has not been sufficient to establish normal ranges for the plasma level of the TIMPs in healthy individuals (Kodama et al, 1989; Clark et al, 1991). Furthermore, recovery of specific signal from clinical samples has not been demonstrated with internal standards.

In a study by Mimori et al (1997) in which tumour tissue levels of TIMP-1 mRNA were studied in patients with gastric carcinoma, high tumour/normal tissue ratios of TIMP-1 mRNA were found to be associated with increased invasion and poor prognosis. ELISA studies of free TIMP-1 in serum from prostate cancer patients (Baker et al, 1994), and TIMP-1 in complex with MMP-9 in plasma from patients with gastrointestinal cancer and gynaecological cancer (Zucker et al, 1995), have demonstrated that the inhibitor, free or in complex, was found in significantly higher levels in blood samples from cancer patients and that patients with high levels of TIMP-1:MMP-9 complex had a shorter survival (Zucker et al, 1995). However, in a study of cervical carcinomas (Nuovo et al, 1995), an increased ratio between expression of MMP-2 and MMP-9 mRNAs and expression of TIMP-1 and TIMP-2 mRNAs was found to be associated with poor survival, suggesting that there may be an excess of MMP activity over TIMP-1 in cancers with a poor prognosis. Similar results were obtained by Gohji et al (1996), who showed that high MMP-2:TIMP-2 protein ratios measured in serum from urothelial cancer patients were related to poor patient outcome.

The goal of the present study was to develop further and validate rigorously one of the previously described TIMP-1 ELISAs when applied to blood samples obtained from healthy donors and cancer patients. Furthermore, this assay was used to

*Received 16 June 1998*
*Revised 28 September 1998*
*Accepted 22 October 1998*

*Correspondence to:* RW Stephens establish reference ranges for TIMP-1 levels in donor plasma. It was shown that healthy donors ($n$ = 194) have a low, and very narrow, range of plasma TIMP-1 levels, while patients with advanced Dukes' stage D colorectal cancer or breast cancer present a diverse scatter of plasma TIMP-1 levels, significantly elevated above the levels found in plasma from blood donors.

### MATERIALS AND METHODS

### Blood donors and patients

Through the cooperation of the blood bank at the Hvidovre University Hospital, Copenhagen, blood samples were initially obtained from 94 volunteer blood donors, comprising 51 males aged 19–59 years (median 41 years) and 43 females aged 20–64 years (median 36 years). In a later collection, 100 donor samples were obtained, comprising 56 males aged 19–59 years (median 42 years) and 44 females aged 20–60 years (median 36.5 years). The donors presented voluntarily at a blood collection centre where normal exclusion criteria applied, and the samples were therefore considered as representing individuals in apparently normal health. In addition, blood was collected from 19 stage IV breast cancer patients (aged 45–70 years) at the Oncology Department, Herlev University Hospital, Copenhagen, and from 143 Dukes' stage D colorectal cancer patients (aged 36–88 years, median 67 years, 60% were male) at the Department of Surgical Gastroenterology, Hvidovre University Hospital, Copenhagen. Informed consent was obtained from all blood donors and patients, and permission was obtained from the local Ethical Committee.

### Blood collections and plasma separation

Peripheral blood was drawn with minimal stasis (if necessary a maximum of 2-min stasis with a tourniquet at maximum +2 kPa was accepted) into prechilled citrate, EDTA, or heparin collection tubes (Becton-Dickinson, Mountain View, CA, USA), mixed by 5 times inversion, and immediately chilled on ice. As soon as possible (no later than 1.5 h after collection) the plasma was separated from blood cells by centrifugation at 4°C at 1200 ***g*** for 30 min, and stored frozen at –80°C prior to assay. Plasma pools were made with freshly collected samples from at least ten donors, aliquoted and stored frozen at –80°C. When analysed, the samples were thawed quickly in a water bath at 37°C and placed on ice until the 1:100 plasma dilutions were made.

### TIMP-1 ELISA

A sensitive and specific sandwich ELISA was developed, using TIMP-1 antibodies developed at the Strangeways Laboratories (Hembry et al, 1985). A sheep polyclonal anti-TIMP-1 antibody (Hembry et al, 1985; Murphy et al, 1991) was used for catching, a murine monoclonal anti-TIMP-1 $IgG_1$ (MAC-15) (Cooksley et al, 1990) for detection of the antigen, and a rabbit anti-mouse immunoglobulins–alkaline phosphatase conjugate (Dako, Glostrup, Denmark) enabled kinetic rate assay. The latter conjugate was supplied preabsorbed against human IgG, thus avoiding cross-reaction with IgG in the plasma samples. As the monoclonal detection antibody MAC-15 recognizes both free TIMP-1 and TIMP-1 in complex with MMPs (Cooksley et al, 1990), the total TIMP-1 content of the measured sample captured by the sheep polyclonal anti-TIMP-1 antibody (Hembry et al, 1985) was determined by the ELISA.

Immunoassay plates (96-well) (Maxisorp, Nunc, Roskilde, Denmark) were coated for 1 h at 37°C with 100 µl per well of polyclonal sheep anti-TIMP-1 (4 mg $l^{-1}$) in 0.1 mol $l^{-1}$ carbonate buffer, pH 9.5. Then the assay wells were rinsed twice with 200 µl per well of SuperBlock™ solution (Pierce Chemicals, Rockford, IL, USA) diluted 1:1 with phosphate-buffered saline (PBS). The immunoassay plates were stored for up to 14 days at –20°C. On the day of use the plates were thawed at room temperature and washed five times in PBS containing 1 g $l^{-1}$ Tween-20. Wells were then treated for 1 h at 30°C with 100 µl per well of triplicate 1:100 dilutions of plasma made in a sample buffer consisting of 50 m mol $l^{-1}$ phosphate, pH 7.2, 0.1 mol $l^{-1}$ sodium chloride (NaCl), 10 g $l^{-1}$ bovine serum albumin (Fraction V, Boehringer-Mannheim, Penzberg, Germany) and 1 g $l^{-1}$ Tween-20. On every assay plate the first three columns of wells (each column consisting of eight wells) were incubated with a series of standards, consisting of seven serial dilutions in triplicate of purified recombinant human TIMP-1, starting at 10 µg $l^{-1}$ then 5, 2.5, 1.25, 0.625, 0.313 and 0.156 µg $1^{-1}$. Also included on each plate were triplicate blank wells containing only sample dilution buffer, and two sets of triplicate wells of a 1:100 dilution of a control citrate plasma pool; the first set of triplicate plasma pool was added as the first sample to the assay plate and the second set of triplicate plasma pool was added as the last. After TIMP-1 binding, the wells were washed five times, then treated for 1 h at 30°C with 100 µl per well of the purified murine monoclonal anti-TIMP MAC-15 (0.5 mg $l^{-1}$) in sample dilution buffer. After another five washes, the wells were incubated for 1 h at 30°C with 100 µl per well of rabbit anti-mouse immunoglobulins–alkaline phosphatase conjugate diluted 1:2000 in sample dilution buffer. Following five washes with washing solution and three washes with pure water, 100 µl of freshly made p-nitrophenyl phosphate (Sigma, St Louis, MO, USA) substrate solution (1.7 g $l^{-1}$ in 0.1 mol $l^{-1}$ Tris-HCl, pH 9.5, 0.1 mol $l^{-1}$ NaCl, 5 mmol $l^{-1}$ magnesium chloride) was added to each well and the plate was placed in a Ceres 900™ plate reader (Bio-Tek Instruments, Winooski, VT, USA). The yellow colour development at 23°C was monitored automatically, with readings taken at 405 nm against an air blank every 10 min for 60 min. KinetiCalc II software was used to manage the data, calculate the rate of colour change for each well (linear regression analysis) and compute from the rates for the TIMP-1 standards a 4-parameter fitted standard curve, from which the TIMP-1 concentration of each plasma sample was calculated.

### Recovery experiments

The recovery of signal from standard TIMP-1 was measured after addition to 1:100 dilutions of citrate, EDTA or heparin plasma pools. Standard TIMP-1 was added to these plasma pool solutions to give final concentrations in the range 0–10 µg $l^{-1}$. The recovery in each case was calculated from the slope of the line representing TIMP-1 signal as a function of concentration, where 100% recovery was defined as the slope obtained when TIMP-1 was diluted in the sample dilution buffer.

### Immunoblotting

Citrate plasma from a patient with a high level of TIMP-1 in blood (634 µg $l^{-1}$, determined by ELISA) was diluted 1:10 and added to a column of protein A-Sepharose containing polyclonal sheep anti-TIMP-1. Following five times recycling, bound proteins were

 © Cancer Research Campaign 1999

eluted from the column and sodium dodecyl sulphate (SDS)-gel electrophoresis of 50 µl of the resulting eluate was carried out using a 12% acrylamide Ready Gel™ (Bio-Rad). A total of 15 µl of a mixture of low molecular weight (Pharmacia) and high molecular weight markers (Bio-Rad) and 50 µl of TIMP-1 standard (100 µg $l^{-1}$) in Laemmli Sample Buffer™ were also run on the gel. Proteins were transferred electrophoretically from the gel onto a polyvinylidene diflouride membrane (Millipore). The membrane was incubated for 1 h at room temperature with 1% skimmed milk powder in TBS. After washing, the membrane was incubated for 1 h at room temperature with 20 ml of MAC-15 at a concentration of 5 µg $l^{-1}$, followed by washing and incubation for another hour at room temperature with 20 ml of rabbit anti-mouse immunoglobulins–alkaline phosphatase conjugate diluted 1:1000. Finally the membrane was washed and phosphate substrate solution (NBT/BCIP) was added to develop colour.

### RESULTS

### ELISA performance

Development of colour in each well was a linear function of time for all concentrations of TIMP-1 measured in these experiments (Figure 1), with correlation coefficients for the automatically fitted lines typically better than 0.99. The standard curve for the rates plotted against the TIMP-1 concentration consisted of the linear and upper curved regions (over the range 0–5 µg $l^{-1}$) of a sigmoidal curve, and the correlation coefficient for the four-parameter fit was typically better than 0.999 (Figure 2). The rate with no TIMP-1 (read against air) was 1.21 ± 0.15 (mean ± standard deviation (s.d.)) milliabsorbance units per min ($n$ = 29), while the rate with 10 µg $l^{-1}$ standard TIMP-1 was 50.3 ± 6.01 milliabsorbance units per min ($n$ = 29). The limit of detection for the assay, defined as the concentration of TIMP-1 corresponding to a signal 3 s.d. above the mean for the TIMP-1 blank, was 0.089 µg $l^{-1}$ or 13% of the mean of the measured concentration of TIMP-1 in healthy citrate plasma samples diluted 1:100. The intra-assay coefficient of variation for 16 replicates of a control citrate plasma pool measured on the same plate was 5.3%, and the inter-assay coefficient of variation for 29 successive assays of the plasma pool (on different days) was 6.2%. This plasma pool had a TIMP-1 content of 57.8 µg $l^{-1}$, corresponding to the 22nd centile of the plasmas subsequently measured.

### Recovery of recombinant TIMP-1 after dilution in plasma

Specific signal recovery was determined by addition of increasing concentrations of purified TIMP-1 standard to a fixed 1:100 dilution of plasma pool and subsequent measurement of the ELISA signal. In diluted citrate plasma pool 104% recovery was obtained, 101% in diluted EDTA plasma pool and 87% in diluted heparin plasma pool (Figure 3). Thus, the recovery of TIMP-1 signal from an internal standard was acceptable for all preparations of plasma, but recovery from EDTA and citrate plasma was more complete than heparin plasma.

### Dilution curves for plasma TIMP-1 signal

Serial dilutions of citrate, EDTA and heparin plasma pools were made and TIMP-1 levels assayed to test for linear reduction in

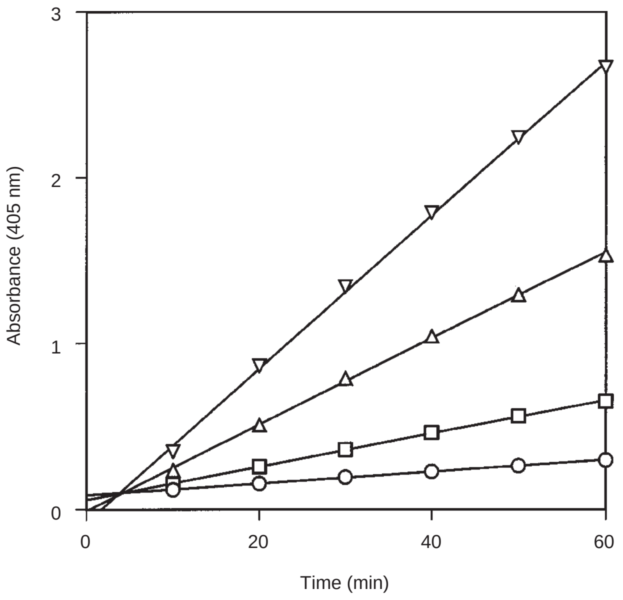

**Figure 1** Kinetic ELISA for TIMP-1. Progress curves for the change in absorbance at 405 nm produced by hydrolysis of p-nitrophenyl phosphate by solid-phase bound alkaline phosphatase immunoconjugate. The data shown were generated by four individual assay wells treated with four different concentrations of purified recombinant TIMP-1; 10 µg $l^{-1}$ (▽), 2.5 µg $l^{-1}$ (△), 0.63 µg $l^{-1}$ (□) and 0.16 µg $l^{-1}$ (○). The lines shown have been fitted by simple linear regression

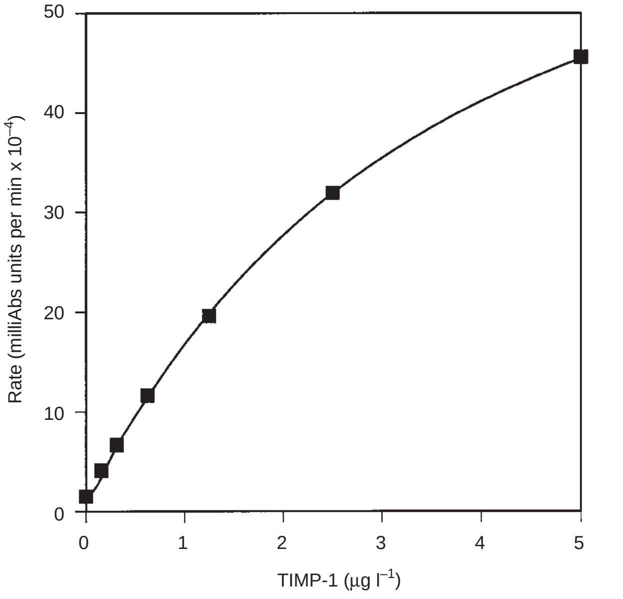

**Figure 2** TIMP-1 standard curve. ELISA-well absorbance measurements for triplicate TIMP-1 standards in the range 0.0–5 µg $1^{-1}$ were collected automatically over 60 min, with readings taken at 405 nm every 10 min. Progress curves were computed for each assay well and the rates thus obtained were fitted to a standard curve using a four-parameter equation of the form $y = d + [(a-d)/(1 + (x/c)b)]$. In the example shown, the four derived parameters had the following values: a = 1.87, b = 1.11, c = 3.35, d = 73.5. The correlation coefficient for the fitted curve was > 0.999

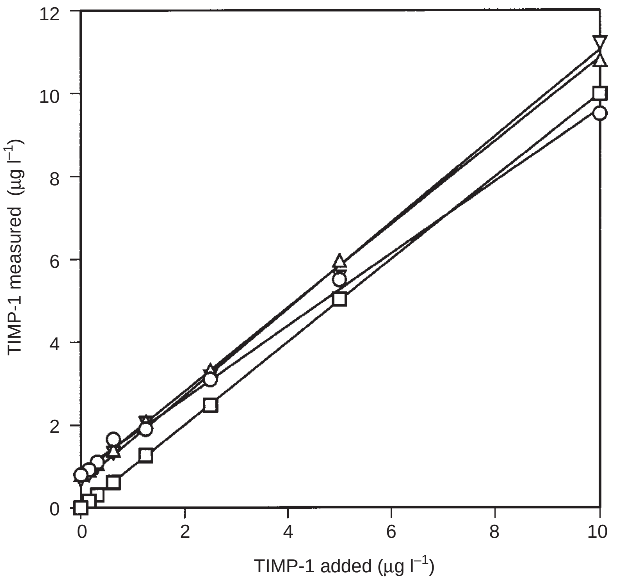

**Figure 3** Recovery of ELISA signal from standard TIMP-1 added in increasing concentration to assay dilution buffer (□), a 1:100 dilution of EDTA plasma pool (△), a 1:100 dilution of citrate plasma pool (▽) and a 1:100 dilution of heparin plasma pool (○). The values shown are the means of triplicates. The correlation coefficient for each fitted curve was greater than 0.99

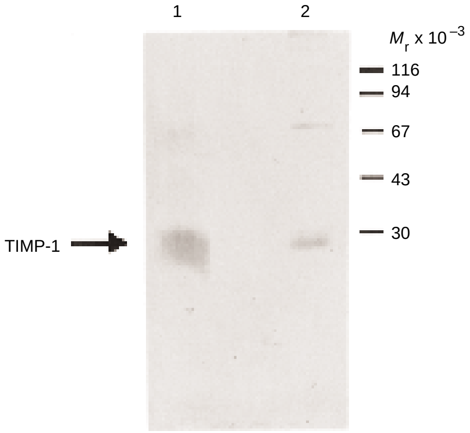

**Figure 4** Western blotting of immunoabsorbed patient plasma sample. Lane 1: standard TIMP-1; lane 2: eluate of patient citrate plasma sample diluted 1:10 and immunoabsorbed with sheep polyclonal anti-TIMP-1. Bands of unreduced standard TIMP-1 and TIMP-1 isolated from plasma sample both appear just below 30 kDa.

ELISA signal. Citrate, EDTA and heparin plasmas all gave good linearity of signal as a function of dilution. The 1% plasma dilution that was chosen for subsequent determinations lay well within the range of this linear dilution curve.

### Immunoblotting of plasma TIMP-1

The Western blot of the immunoabsorbed patient plasma sample showed a clear band of 28 kDa (Figure 4, lane 2), corresponding to free, uncomplexed TIMP-1 (Figure 4, lane 1). No bands were found at the expected higher molecular weights corresponding to complexes between MMPs and TIMP-1, e.g. MMP-2:TIMP-1, 100 kDa. This could indicate either that the majority of TIMP-1 was present in the plasma as the free form, or that complexes were dissociated during SDS polyacrylamide gel electrophoresis (SDS-PAGE). Although the sample was left both unreduced and unheated in order to preserve any complexes present in the plasma sample, it has been reported that MMP:TIMP complexes may be unstable in SDS-PAGE (Stetler-Stevenson et al, 1989; Wilhelm et al, 1989; Moll et al, 1990), even under non-reducing SDS-PAGE conditions (Moutsiakis et al, 1992).

### TIMP-1 in citrate and EDTA plasma from the same healthy donors

A collection of citrate and EDTA plasma samples taken simultaneously from 100 healthy donors was available for this study. These samples were not specifically collected as platelet-poor plasma. However, when seven blood samples were prepared both as platelet-poor plasma and as protocol plasma, no significant difference was found in their TIMP-1 levels (data not shown), so that platelet TIMP-1 contamination of plasma in the present study was considered insignificant. The percentile plots for TIMP-1 levels determined in these samples are shown in Figure 5A. The values in each set approximated a normal distribution; the citrate plasma TIMP-1 levels had a reference range (10th to 90th centile) of 55.0–90.3 μg $l^{-1}$ and a mean of 69.2 ± 13.1 μg $l^{-1}$. Similarly, the reference range for the EDTA plasma TIMP-1 levels was from 58.0 to 91.8 μg $l^{-1}$ and the mean was 73.5 ± 14.2 μg $l^{-1}$. For both citrate and EDTA plasma the mean TIMP-1 levels lay in close proximity to the median levels (Table 1). A paired means comparison showed that the level of TIMP-1 in citrate plasma was significantly lower by 4.34 μg $l^{-1}$ (95% confidence interval (CI) 2.34–6.33; $P < 0.0001$) than the level in EDTA plasma from the same individual. However, it is likely that this reflected the difference in sampling procedure when collecting EDTA and citrate plasma from the donors. EDTA plasma tubes contained dry anticoagulant material, while citrate plasma tubes contained a small amount of liquid citrate buffer which gave a small and variable systematic dilution error (× 9/10). The level of TIMP-1 in citrate plasma correlated with EDTA plasma from the same individuals: the linear regression plot in Figure 5B shows a regression coefficient of 0.99 and the slope of the fitted line is 0.93, illustrating the small dilution error. A non-parametric Spearman's rank test for the data set gave a rho value of 0.62 and $P < 0.0001$.

### TIMP-1 levels in citrate plasma

In total, 194 citrate plasma samples from healthy blood donors were assayed, comprising 94 samples taken from one collection and 100 samples taken 9 months later from a different set of donors. Figure 6 shows the percentile plots for TIMP-1 levels measured in these two independent groups. The reference range (10th to 90th centile) for TIMP-1 levels in citrate plasma from the first collection was 53.3–77.7 μg $l^{-1}$ with a mean TIMP-1 level of 65.4 ± 10.1 μg $l^{-1}$ which was indistinguishable from the median (Table 1); the values approximating a normal distribution. The mean TIMP-1 level for the second collection was 69.2 ± 13.1 μg $l^{-1}$ (reference range 55.0–90.3 μg $l^{-1}$). An unpaired means comparison showed that TIMP-1 levels measured in the two sets of citrate

© Cancer Research Campaign 1999

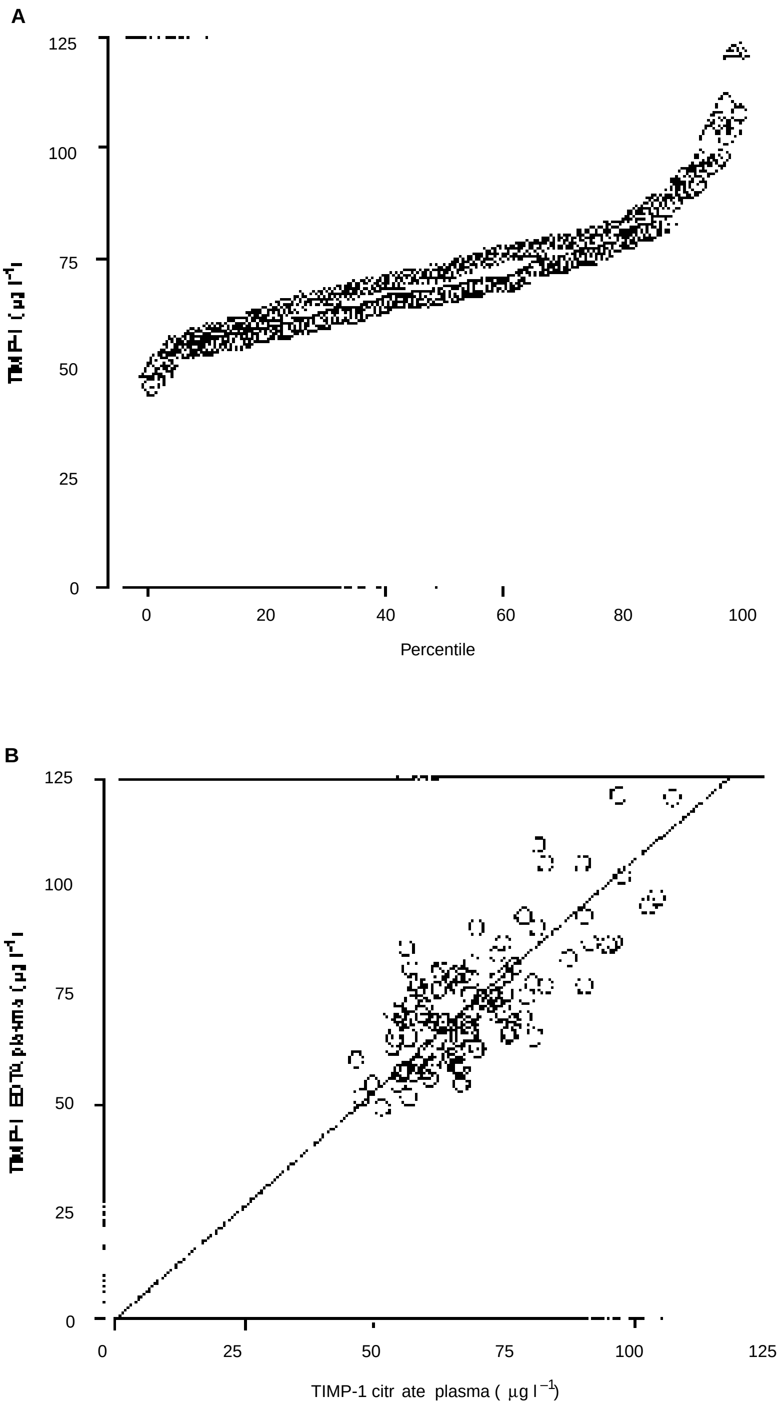

**Figure 5** (**A**) Percentile plot for the level of TIMP-1 (µg l–1) measured by ELISA in citrate plasma (○) and EDTA plasma (△) from the same individual in a set of 100 volunteer blood donors. (**B**) Linear regression plot for the level of TIMP-1 in citrate plasma samples compared with EDTA plasma samples from the same 100 individuals. The equation of the fitted line is y = 0.93x, with a regression coefficient of 0.99

plasma samples taken in the two different collections differed only by 3.82 µg $l^{-1}$ (95% CI 0.50–7.14 µg $l^{-1}$; $P = 0.024$). Moreover, no significant difference was apparent between the plasma pool controls ($n = 8$) included in each set of assays (mean difference 0.36 µg $l^{-1}$; 95% CI – 1.71–2.44; $P = 0.69$). The mean TIMP-1 level for the total material of 194 citrate plasma samples was 67.3 ± 11.8 µg $l^{-1}$, which was close to the median of 66.1 µg $l^{-1}$, the levels approximating a normal distribution (reference range 54.0–82.7 µg $l^{-1}$).

### Tests for correlations to gender and age of the donor

In the series of assays performed to obtain these results, the interassay variation for the control plasma value was 2.7%.

© Cancer Research Campaign 1999

**Table 1** Summary of TIMP-1 levels found in blood from healthy donors

| Blood fraction | Date of sampling | Number of samples | Mean ± s.d. (μg l$^{-1}$) | Median (μg l$^{-1}$) | Reference range[a] (μg l$^{-1}$) |
|---|---|---|---|---|---|
| Citrate plasma | Sept 1996 | 94 | 65.4 ± 10.1 | 65.6 | 53.3–77.7 |
| Citrate plasma | May 1997 | 100[b] | 69.2 ± 13.1 | 67.0 | 55.0–90.3 |
| Citrate plasma | 1996 + 1997 | 194 | 67.3 ± 11.8 | 66.1 | 54.0–82.7 |
| EDTA plasma | May 1997 | 100[b] | 73.5 ± 14.2 | 71.2 | 58.0–91.8 |

[a]The reference range is defined as between the 10th and 90th percentiles. [b]These samples were collected from the same donors.

Percentiles for TIMP-1 levels in 194 citrate plasma samples divided according to gender are shown in Figure 7. The mean TIMP-1 value for 107 male donors was 70.4 ± 12.0 μg l$^{-1}$ (median 69.4 μg l$^{-1}$) with a reference range (10th to 90th centile) from 56.2 to 86.6 μg l$^{-1}$, while the mean TIMP-1 value for 87 female donors in this set was 63.5 ± 10.5 μg l$^{-1}$ (median 62.0 μg l$^{-1}$) with the reference range from 51.8 to 77.0 μg l$^{-1}$. There was a significant difference ($P < 0.0001$) in TIMP-1 mean levels between the two groups; males were higher by 6.91 μg l$^{-1}$ than females (95% CI 3.67–10.14 μg l$^{-1}$, unpaired means comparison). There was a relatively weak association between plasma TIMP-1 and age (Spearman's rho = 0.33), which was significant ($P = 0.0011$). This did not show any gender specificity, and was weak for both females (Spearman's rho = 0.29, $P = 0.006$) and males (Spearman's rho = 0.35, $P = 0.0003$). In the EDTA plasma donor material the mean TIMP-1 value for 56 males was 76.9 ± 15.0 μg l$^{-1}$ (median 75.1 μg l$^{-1}$) with a reference range from 58.8 to 96.9 μg 1$^{-1}$, while 44 female donors had a mean TIMP-1 plasma level of 69.3 ± 11.8 μg l$^{-1}$ (median 67.9) with a reference range from 56.1 to 85.5 μg l$^{-1}$. Again, a significant difference in TIMP-1 means appeared between males and females, with males higher than females by 7.53 μg l$^{-1}$ (95% CI 2.04–13.0; $P = 0.0076$, unpaired means comparison).

### TIMP-1 levels in plasma from patients with advanced cancer

TIMP-1 was measured in EDTA plasma samples from 143 patients with Dukes' stage D colorectal cancer, and a mean TIMP-1 level of 240 ± 145 μg l$^{-1}$ and a median value of 193 μg 1$^{-1}$ were found (reference range 106–451 μg l$^{-1}$). Compared to EDTA plasma samples from 100 healthy donors, a highly significant difference in TIMP-1 plasma levels ($P < 0.0001$, Mann–Whitney) was apparent between colorectal cancer patients and healthy donors. Ninety per cent of the colorectal cancer patients in this study had TIMP-1 plasma values that were above the 95th centile of TIMP-1 levels in donor plasma samples (Figure 8). While the cancer patients, especially those with colorectal cancer, were older than the control populations, the increase in TIMP-1 with age is considered too weak to account for the greatly increased levels found in the cancer patients. In fact, the correlation between TIMP-1 level and age of the colorectal cancer patients was weaker (rho = 0.18) than for the donors (rho = 0.33), indicating that their disease was the dominant factor contributing to the increase in their plasma level of TIMP-1.

In a pilot study including 19 breast cancer patients with stage IV disease, TIMP-1 levels in patient and healthy female donor citrate

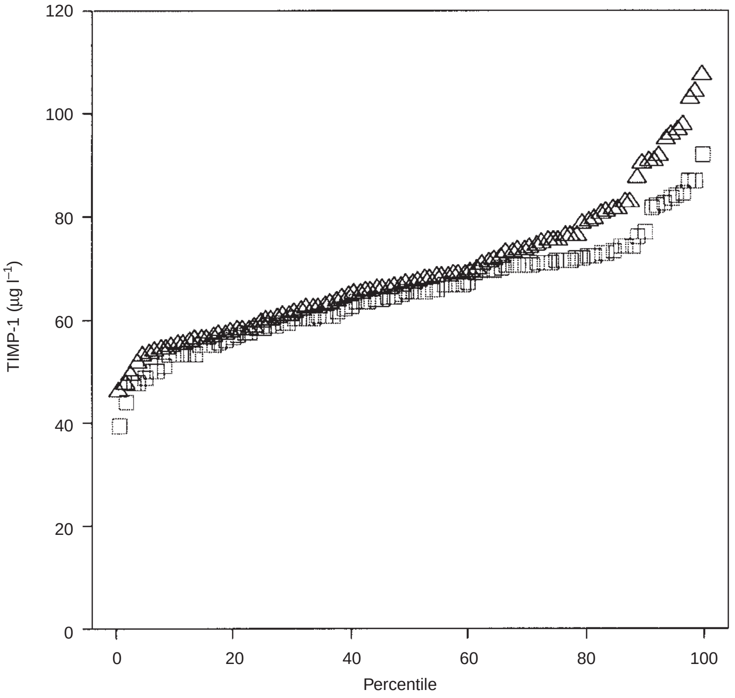

**Figure 6** Percentile plot for the level of TIMP-1 (μg l$^{-1}$) measured by ELISA in two sets of citrate plasma samples obtained by the same procedure from volunteer blood donors at different times: 100 samples from May 1997 (△) and 94 samples from Sept 1996 (□)

© Cancer Research Campaign 1999

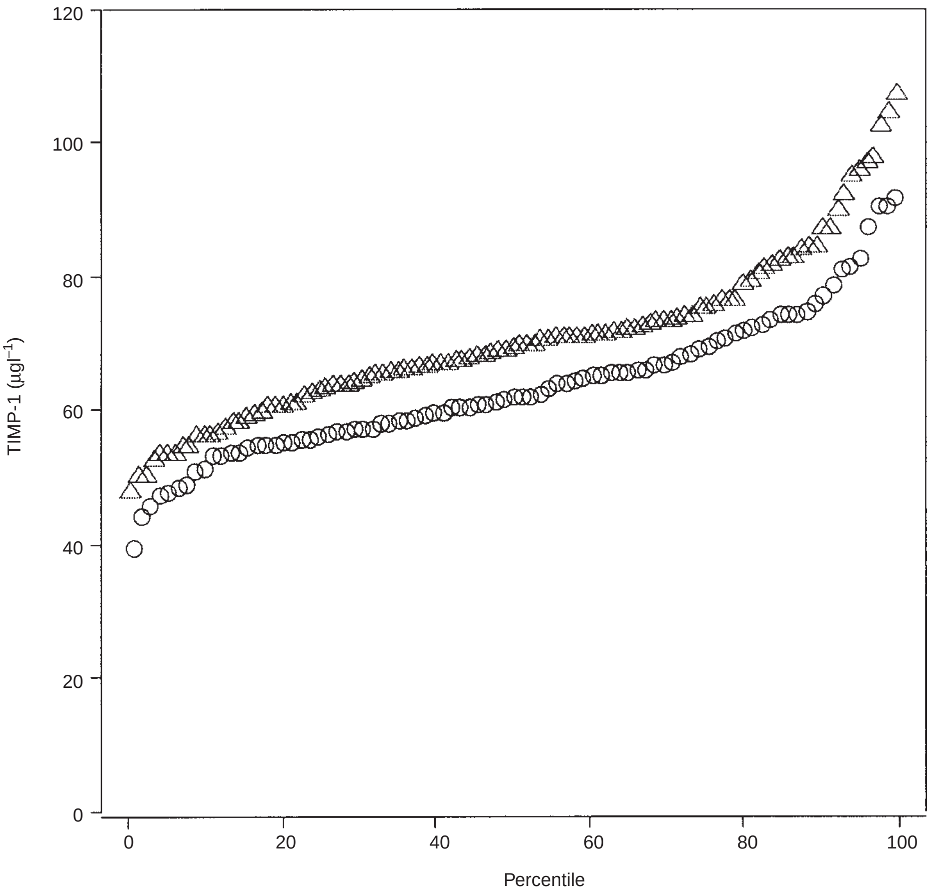

**Figure 7** Percentile plot for the level of TIMP-1 (µg $l^{-1}$) measured by ELISA in 194 citrate plasma samples from volunteer blood donors and divided by gender into 107 males (△) and 87 females (○)

plasma were compared. The mean TIMP-1 level measured in the citrate plasma samples from the 19 breast cancer patients was 292 ± 331 µg $l^{-1}$ (median 236 µg $l^{-1}$) compared to a mean TIMP-1 level of 63.5 ± 10.5 µg $l^{-1}$ (median 62.0 µg $l^{-1}$) in citrate plasma samples from 87 healthy female donors with a reference range of 51.8–77.0 µg $l^{-1}$. Bearing in mind the limited amount of patient data in this comparison, a Wald–Wolfowitz runs test indicated a highly significant difference with $P < 0.0001$ between patient TIMP-1 levels and those of healthy donors.

### DISCUSSION

The assay described above enabled accurate determination of total TIMP-1 in human plasma samples. Detection of captured TIMP-1 with MAC-15 was conveniently followed by incubation with a rabbit anti-mouse immunoglobulins–alkaline phosphatase conjugate, which allowed kinetic rate assays of the bound antigen. This permitted automated fitting of rate curves which has proven considerably more reliable than single end point measurements. The use of a rapid blocking agent and a dilution buffer with high buffering capacity also facilitated reproducible assays. Including these elements in the final assay, requirements of sensitivity, specificity, stability and good recovery of an internal standard were fulfilled.

The quantitative studies of TIMP-1 in blood from healthy donors showed that both citrate and EDTA plasma samples are suitable for the ELISA determination. Compared to other published ELISA studies of TIMP-1 in healthy donors' plasma (Fung et al, 1996; Jung et al, 1996), the levels of TIMP-1 found in the present study fell within a very narrow range. Some studies have reported values for serum, but plasma was selected for the present study to avoid the variable contribution of platelet activation to the measured TIMP-1 values (Cooper et al, 1985). While the plasma samples used in this study were not specifically prepared as platelet-poor plasma we do not anticipate, from tests we have done, that this would significantly change the values measured. The donor material was large enough to show that TIMP-1 levels in healthy plasma (both EDTA and citrate) approximated a normal distribution, for females as well as for males. For both EDTA and citrate plasma, the mean TIMP-1 levels were approximately 10% higher in males than in females. It could be speculated that the reason for these higher levels of inhibitor in the blood of males is a higher release rate of TIMP-1 into blood from activated platelets, reflecting a tendency towards higher incidence of thromboembolic disease amongst the male population. When the males and females were considered separately, there was a weak correlation between TIMP-1 level and age as seen for the whole population (see above).

The remodelling of tissue which characterizes numerous physiological as well as pathological events, e.g. wound healing, trophoblast invasion, mammary gland involution, malignant tumour invasion and metastasis, is well known to employ the matrix-degrading abilities of the MMPs (Matrisian, 1992; Stetler-Stevenson et al, 1996). Several studies of human cancers have

© Cancer Research Campaign 1999 

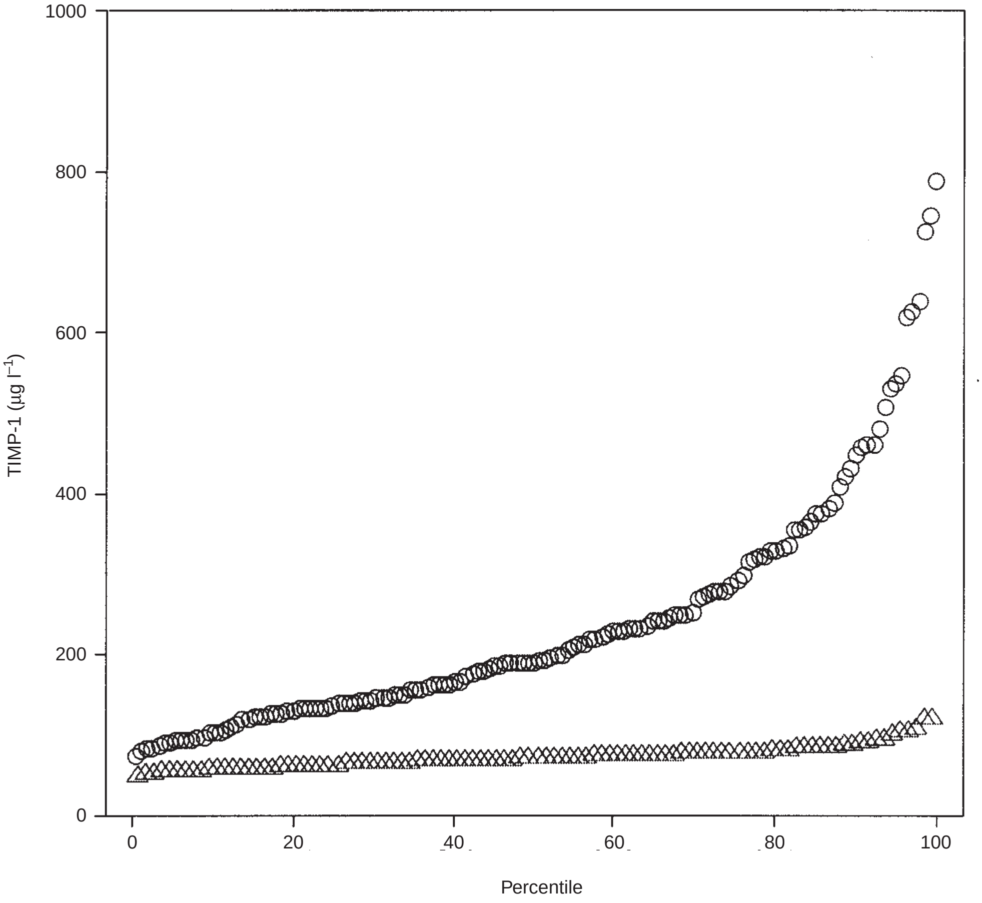

**Figure 8** Plasma TIMP-1 levels (µg $l^{-1}$) determined by ELISA in blood samples from patients with Dukes' stage D colorectal cancer. In 143 EDTA plasma samples from patients with Dukes' stage D disease (○), TIMP-1 levels were measured and compared with levels found in EDTA plasma samples from 100 volunteer blood donors (△)

shown increased expression of MMPs in the tumour tissue (De Nictolis et al, 1996; Kawano et al, 1997), but also increased expression of the inhibitors of MMPs (TIMP-1 and TIMP-2) (Fong, 1996; Yoshiji et al, 1996). ELISA studies of free TIMP-1 in serum from prostate cancer patients have demonstrated that the inhibitor is significantly increased in these patients (Baker et al, 1994). Increased plasma levels of MMP-9:TIMP-1 complex have been found in patients with gastrointestinal cancer and gynaecological cancer (Zucker et al, 1995). These findings could imply that growth and metastatic spread of malignant tumours necessarily involves a higher level of activation of the MMP system with a concomitant or compensatory increase in TIMP expression. In this context, it is noteworthy that TIMP-1 has been found to have a growth-promoting effect on both normal and malignant cells (Hayakawa et al, 1992). Similarly, increased levels of the type 1 inhibitor of plasminogen activator (PAI-1) are now associated with poor prognosis in several types of cancer (Frandsen et al, 1998), suggesting that PAIs, and perhaps also TIMPs may themselves be involved in the progression of cancer through a mechanism independent of their anti-proteolytic activity (Deng et al, 1996).

The ELISA described above was applied to plasma samples from patients with Dukes' stage D colorectal cancer and patients with advanced breast cancer, and it was found that in both cancer types, plasma TIMP-1 was significantly increased as compared to donor plasma levels. It is especially noteworthy that, in the 143 colorectal cancer patients, 90% of the patients had plasma TIMP-1 levels that were above the 95th centile of the plasma TIMP-1 levels in the donor plasmas, suggesting a potential application of plasma TIMP-1 measurements in screening for colorectal cancer. Moreover, since the results from the colorectal cancer and the breast cancer study suggest that increased plasma TIMP-1 levels may be a common feature of patients with advanced cancer, it should therefore be investigated whether plasma TIMP-1 could be useful as a marker of tumour recurrence. Finally, since high TIMP-1 mRNA levels in cancer tissue and high TIMP-1 protein level in plasma have been associated with poor survival of cancer patients, it is suggested that measurement of TIMP-1 in blood of cancer patients be further evaluated for its value as a prognostic marker.

### ACKNOWLEDGEMENTS

We thank the Department of Surgical Gastroenterology at the Hvidovre University Hospital. This work was supported by the Danish Cancer Society, the Danish Cancer Research Fund, the Foundation of 17.12.1981, the Dagmar Marshalls Fund, the Wedell–Wedellsborgs Fund, the Clinical Research Unit, Department of Oncology, Herlev Hospital, the Ingeborg Roikjers Fund, the Pharmacy Fund of 1991, the Einar and Vera Hansens Fund, and the University of Copenhagen.

© Cancer Research Campaign 1999

### REFERENCES

Baker T, Tickle S, Wasan H, Docherty A, Isenberg D and Waxman J (1994) Serum metalloproteinases and their inhibitors: markers for malignant potential. *Br J Cancer* **70**: 506–512

Birkedal-Hansen H, Moore WG, Bodden MK, Windsor LJ, Birkedal-Hansen B, DeCarlo A and Engler JA (1993) Matrix metalloproteinases: a review. *Crit Rev Oral Biol Med* **4**: 197–250

Clark IM, Powell LK, Wright JK and Cawston the (1991) Polyclonal and monoclonal antibodies against human tissue inhibitor of metalloproteinases (TIMP) and the design of an enzyme-linked immunosorbent assay to measure TIMP. *Matrix* **11**: 76–85

Cooksley S, Hipkiss JB, Tickle SP, Holmes IE, Docherty AJ, Murphy G and Lawson AD (1990) Immunoassays for the detection of human collagenase, stromelysin, tissue inhibitor of metalloproteinases (TIMP) and enzyme-inhibitor complexes. *Matrix* **10**: 285–291

Cooper TW, Eisen AZ, Stricklin GP and Welgus HG (1985) Platelet-derived collagenase inhibitor: characterization and subcellular localization. *Proc Natl Acad Sci USA* **82**: 2779–2783

De Nictolis M, Garbisa S, Lucarini G, Goteri G, Masiero L, Ciavattini A, Garzetti GG, Stetler-Stevenson WG, Fabris G, Biagini G and Prat J (1996) 72-kilodalton type IV collagenase, type IV collagen, and Ki 67 antigen in serous tumors or the ovary: a clinicopathologic, immunohistochemical, and serological study. *Int J Gynecol Pathol* **15**: 102–109

DeClerck YA, Perez N, Shimada H, Boone TC, Langley KE and Taylor SM (1992) Inhibition of invasion and metastasis in cells transfected with an inhibitor of metalloproteinases. *Cancer Res* **52**: 701–708

Deng G, Curriden SA, Wang S, Rosenberg S and Loskutoff DJ (1996) Is plasminogen activator inhibitor-1 the molecular switch that governs urokinase receptor-mediated cell adhesion and release? *J Cell Biol* **134**: 1563–1571

Fong KM (1996) TIMP1 and adverse prognosis in non-small cell lung cancer. *Clin Cancer Res* **2**: 1369–1372

Frandsen TL, Stephens RW, Pedersen AN, Engelholm LH, Holst-Hansen C and Brünner N (1998) Plasminogen activator inhibitor type 1 (PAI-1) in cancer: a potential new target for antiinvasive and antimetastatic therapy. *Drugs of the Future* **23**: 873–883

Fujimoto N, Zhang J, Iwata K, Shinya T, Okada Y and Hayakawa T (1993) A one-step sandwich enzyme immunoassay for tissue inhibitor of metalloproteinases-2 using monoclonal antibodies. *Clin Chim Acta* **220**: 31–45

Fung K, Nowak L, Lein M, Henke W, Schnorr D and Loening SA (1996) Role of specimen collection in preanalytical variation of metalloproteinases and their inhibitors in blood. *Clin Chem* **42**: 2043–2045

Gohji K, Fujimoto N, Fujii A, Komiyama T, Okawa J and Nakajima M (1996) Prognostic significance of circulating matrix metalloproteinase-2 to tissue inhibitor of metalloproteinases-2 ratio in recurrence of urothelial cancer after complete resection. *Cancer Res* **56**: 3196–3198

Goldberg GI, Strongin A, Collier IE, Genrich LT and Marmer BL (1992) Interaction of 92-kDa type IV collagenase with the tissue inhibitor of metalloproteinases prevents dimerization, complex formation with interstitial collagenase, and activation of the proenzyme with stromelysin. *J Biol Chem* **267**: 4583–4591

Hayakawa T, Yamashita K, Tanzawa K, Uchijima E and Iwata K (1992) Growth-promoting activity of tissue inhibitor of metalloproteinases-1 (TIMP-1) for a wide range of cells. A possible new growth factor in serum. *FEBS Lett* **298**: 29–32

Hembry RM, Murphy G and Reynolds JJ (1985) Immunolocalization of tissue inhibitor of metalloproteinases (TIMP) in human cells. Characterization and use of a specific antiserum. *J Cell Sci* **73**: 105–119

Jung K, Nowak L, Lein M, Henke W, Schnorr D and Loening SA (1996) What kind of specimen should be selected for determining tissue inhibitor of metalloproteinase-1 (TIMP-1) in blood? *Clin Chim Acta* **254**: 97–100

Kawano N, Osawa H, Ito T, Nagashima Y, Hirahara F, Inayama Y, Nakatani Y, Kimura S, Kitajima H, Koshikawa N, Miyazaki K and Kitamura H (1997) Expression of gelatinase A, tissue inhibitor of metalloproteinases-2, matrilysin, and trypsin(ogen) in lung neoplasms: an immunohistochemical study. *Hum Pathol* **28**: 613–622

Khokha R and Waterhouse P (1993) The role of tissue inhibitor of metalloproteinase-1 in specific aspects of cancer progression and reproduction. *J Neurooncol* **18**: 123–127

Khokha R, Zimmer MJ, Graham CH, Lala PK and Waterhouse P (1992*a*) Suppression of invasion by inducible expression of tissue inhibitor of metalloproteinase-1 (TIMP-1) in B16-F10 melanoma cells. *J Natl Cancer Inst* **84**: 1017–1022

Khokha R, Zimmer MJ, Wilson SM and Chambers AF (1992*b*) Up-regulation of TIMP-1 expression in B16-F10 melanoma cells suppresses their metastatic ability in chick embryo. *Clin Exp Metastasis* **10**: 365–370

Kleiner Jr DE, Tuuttila A, Tryggvason K and Stetler-Stevenson WG (1993) Stability analysis of latent and active 72-kDa type IV collagenase: the role of tissue inhibitor of metalloproteinases-2 (TIMP-2). *Biochemistry* **32**: 1583–1592

Kodama S, Yamashita K, Kishi J, Iwata K and Hayakawa T (1989) A sandwich enzyme immunoassay for collagenase inhibitor using monoclonal antibodies. *Matrix* **9**: 1–6

Liotta LA, Steeg PS and Stetler-Stevenson WG (1991) Cancer metastasis and angiogenesis: an imbalance of positive and negative regulation. *Cell* **64**: 327–336

MacDougall JR and Matrisian LM (1995) Contributions of tumor and stromal matrix metalloproteinases to tumor progression, invasion and metastasis. *Cancer Metastasis Rev* **14**: 351–362

Matrisian LM (1992) The matrix-degrading metalloproteinases. *Bioessays* **14**: 455–463

Mimori K, Mori M, Shiraishi T, Fujie T, Baba K, Haraguchi M, Abe R, Ueo H and Akiyoshi T (1997) Clinical significance of tissue inhibitor of metalloproteinase expression in gastric carcinoma. *Br J Cancer* **76**: 531–536

Moll UM, Youngleib GL, Rosinski KB and Quigley JP (1990) Tumor promoter-stimulated $M_r$ 92,000 gelatinase secreted by normal and malignant human cells: isolation and characterization of the enzyme from HT1080 tumor cells. *Cancer Res* **50**: 6162–6170

Moutsiakis D, Mancuso P, Krutzsch H, Stetler-Stevenson WG and Zucker S (1992) Characterization of metalloproteinases and tissue inhibitors of metalloproteinases in human plasma. *Connect Tissue Res* **28**: 213–230

Murphy G, Houbrechts A, Cockett MI, Williamson RA, O'Shea M and Docherty AJ (1991) The N-terminal domain of tissue inhibitor of metalloproteinases retains metalloproteinase inhibitory activity. *Biochemistry* **30**: 8097–8102

Nuovo GJ, MacConnell PB, Simsir A, Valea F and French DL (1995) Correlation of the in situ detection of polymerase chain reaction-amplified metalloproteinase complementary DNAs and their inhibitors with prognosis in cervical carcinoma. *Cancer Res* **55**: 267–275

Stetler-Stevenson WG, Krutzsch HC and Liotta LA (1989) Tissue inhibitor of metalloproteinase (TIMP-2). A new member of the metalloproteinase inhibitor family. *J Biol Chem* **264**: 17374–17378.

Stetler-Stevenson WG, Liotta LA and Kleiner Jr DE (1993) Extracellular matrix 6: role of matrix metalloproteinases in tumor invasion and metastasis. *FASEB J* **7**: 1434–1441

Stetler-Stevenson WG, Hewitt R and Corcoran M (1996) Matrix metalloproteinases and tumor invasion: from correlation and causality to the clinic. *Semin Cancer Biol* **7**: 147–154

Thorgeirsson UP, Lindsay CK, Cottam DW and Gomez DE (1993) Tumor invasion, proteolysis, and angiogenesis. *J Neurooncol* **18**: 89–103.

Welgus HG, Jeffrey JJ, Eisen AZ, Roswit WT and Stricklin GP (1985) Human skin fibroblast collagenase: interaction with substrate and inhibitor. *Coll Relat Res* **5**: 167–179

Wilhelm SM, Collier IE, Marmer BL, Eisen AZ, Grant GA and Goldberg GI (1989) SV40-transformed human lung fibroblasts secrete a 92-kDa type IV collagenase which is identical to that secreted by normal human macrophages *J Biol Chem* **264**: 17213–17221

Yoshiji H, Gomez DE and Thorgeirsson UP (1996) Enhanced RNA expression of tissue inhibitor of metalloproteinases-1 (TIMP-1) in human breast cancer. *Int J Cancer* **69**: 131–134

Zucker S, Lysik RM, DiMassimo BI, Zarrabi HM, Moll UM, Grimson R, Tickle SP and Docherty AJ (1995) Plasma assay of gelatinase B: tissue inhibitor of metalloproteinase complexes in cancer. *Cancer* **76**: 700–708

© Cancer Research Campaign 1999

## References

[PDF_00617] Baker T., Tickle S., Wasan H., Docherty A., Isenberg D., Waxman J. (1994). Serum metalloproteinases and their inhibitors: markers for malignant potential.. Br J Cancer.

[PDF_00620] Birkedal-Hansen H., Moore W. G., Bodden M. K., Windsor L. J., Birkedal-Hansen B., DeCarlo A., Engler J. A. (1993). Matrix metalloproteinases: a review.. Crit Rev Oral Biol Med.

[PDF_00623] Clark I. M., Powell L. K., Wright J. K., Cawston T. E. (1991). Polyclonal and monoclonal antibodies against human tissue inhibitor of metalloproteinases (TIMP) and the design of an enzyme-linked immunosorbent assay to measure TIMP.. Matrix.

[PDF_00627] Cooksley S., Hipkiss J. B., Tickle S. P., Holmes-Ievers E., Docherty A. J., Murphy G., Lawson A. D. (1990). Immunoassays for the detection of human collagenase, stromelysin, tissue inhibitor of metalloproteinases (TIMP) and enzyme-inhibitor complexes.. Matrix.

[PDF_00631] Cooper T. W., Eisen A. Z., Stricklin G. P., Welgus H. G. (1985). Platelet-derived collagenase inhibitor: characterization and subcellular localization.. Proc Natl Acad Sci U S A.

[PDF_00634] De Nictolis M., Garbisa S., Lucarini G., Goteri G., Masiero L., Ciavattini A., Garzetti G. G., Stetler-Stevenson W. G., Fabris G., Biagini G. (1996). 72-kilodalton type IV collagenase, type IV collagen, and Ki 67 antigen in serous tumors or the ovary: a clinicopathologic, immunohistochemical, and Serological study.. Int J Gynecol Pathol.

[PDF_00639] DeClerck Y. A., Perez N., Shimada H., Boone T. C., Langley K. E., Taylor S. M. (1992). Inhibition of invasion and metastasis in cells transfected with an inhibitor of metalloproteinases.. Cancer Res.

[PDF_00642] Deng G., Curriden S. A., Wang S., Rosenberg S., Loskutoff D. J. (1996). Is plasminogen activator inhibitor-1 the molecular switch that governs urokinase receptor-mediated cell adhesion and release?. J Cell Biol.

[PDF_00645] Fong K. M., Kida Y., Zimmerman P. V., Smith P. J. (1996). TIMP1 and adverse prognosis in non-small cell lung cancer.. Clin Cancer Res.

[PDF_00651] Fujimoto N., Zhang J., Iwata K., Shinya T., Okada Y., Hayakawa T. (1993). A one-step sandwich enzyme immunoassay for tissue inhibitor of metalloproteinases-2 using monoclonal antibodies.. Clin Chim Acta.

[PDF_00657] Gohji K., Fujimoto N., Fujii A., Komiyama T., Okawa J., Nakajima M. (1996). Prognostic significance of circulating matrix metalloproteinase-2 to tissue inhibitor of metalloproteinases-2 ratio in recurrence of urothelial cancer after complete resection.. Cancer Res.

[PDF_00661] Goldberg G. I., Strongin A., Collier I. E., Genrich L. T., Marmer B. L. (1992). Interaction of 92-kDa type IV collagenase with the tissue inhibitor of metalloproteinases prevents dimerization, complex formation with interstitial collagenase, and activation of the proenzyme with stromelysin.. J Biol Chem.

[PDF_00665] Hayakawa T., Yamashita K., Tanzawa K., Uchijima E., Iwata K. (1992). Growth-promoting activity of tissue inhibitor of metalloproteinases-1 (TIMP-1) for a wide range of cells. A possible new growth factor in serum.. FEBS Lett.

[PDF_00669] Hembry R. M., Murphy G., Reynolds J. J. (1985). Immunolocalization of tissue inhibitor of metalloproteinases (TIMP) in human cells. Characterization and use of a specific antiserum.. J Cell Sci.

[PDF_00654] Jung K., Nowak L., Lein M., Henke W., Schnorr D., Loening S. A. (1996). Role of specimen collection in preanalytical variation of metalloproteinases and their inhibitors in blood.. Clin Chem.

[PDF_00672] Jung K., Nowak L., Lein M., Henke W., Schnorr D., Loening S. A. (1996). What kind of specimen should be selected for determining tissue inhibitor of metalloproteinase-1 (TIMP-1) in blood?. Clin Chim Acta.

[PDF_00675] Kawano N., Osawa H., Ito T., Nagashima Y., Hirahara F., Inayama Y., Nakatani Y., Kimura S., Kitajima H., Koshikawa N. (1997). Expression of gelatinase A, tissue inhibitor of metalloproteinases-2, matrilysin, and trypsin(ogen) in lung neoplasms: an immunohistochemical study.. Hum Pathol.

[PDF_00680] Khokha R., Waterhouse P. (1994). The role of tissue inhibitor of metalloproteinase-1 in specific aspects of cancer progression and reproduction.. J Neurooncol.

[PDF_00683] Khokha R., Zimmer M. J., Graham C. H., Lala P. K., Waterhouse P. (1992). Suppression of invasion by inducible expression of tissue inhibitor of metalloproteinase-1 (TIMP-1) in B16-F10 melanoma cells.. J Natl Cancer Inst.

[PDF_00687] Khokha R., Zimmer M. J., Wilson S. M., Chambers A. F. (1992). Up-regulation of TIMP-1 expression in B16-F10 melanoma cells suppresses their metastatic ability in chick embryo.. Clin Exp Metastasis.

[PDF_00690] Kleiner D. E., Tuuttila A., Tryggvason K., Stetler-Stevenson W. G. (1993). Stability analysis of latent and active 72-kDa type IV collagenase: the role of tissue inhibitor of metalloproteinases-2 (TIMP-2).. Biochemistry.

[PDF_00693] Kodama S., Yamashita K., Kishi J., Iwata K., Hayakawa T. (1989). A sandwich enzyme immunoassay for collagenase inhibitor using monoclonal antibodies.. Matrix.

[PDF_00696] Liotta L. A., Steeg P. S., Stetler-Stevenson W. G. (1991). Cancer metastasis and angiogenesis: an imbalance of positive and negative regulation.. Cell.

[PDF_00699] MacDougall J. R., Matrisian L. M. (1995). Contributions of tumor and stromal matrix metalloproteinases to tumor progression, invasion and metastasis.. Cancer Metastasis Rev.

[PDF_00702] Matrisian L. M. (1992). The matrix-degrading metalloproteinases.. Bioessays.

[PDF_00704] Mimori K., Mori M., Shiraishi T., Fujie T., Baba K., Haraguchi M., Abe R., Ueo H., Akiyoshi T. (1997). Clinical significance of tissue inhibitor of metalloproteinase expression in gastric carcinoma.. Br J Cancer.

[PDF_00707] Moll U. M., Youngleib G. L., Rosinski K. B., Quigley J. P. (1990). Tumor promoter-stimulated Mr 92,000 gelatinase secreted by normal and malignant human cells: isolation and characterization of the enzyme from HT1080 tumor cells.. Cancer Res.

[PDF_00712] Moutsiakis D., Mancuso P., Krutzsch H., Stetler-Stevenson W., Zucker S. (1992). Characterization of metalloproteinases and tissue inhibitors of metalloproteinases in human plasma.. Connect Tissue Res.

[PDF_00715] Murphy G., Houbrechts A., Cockett M. I., Williamson R. A., O'Shea M., Docherty A. J. (1991). The N-terminal domain of tissue inhibitor of metalloproteinases retains metalloproteinase inhibitory activity.. Biochemistry.

[PDF_00718] Nuovo G. J., MacConnell P. B., Simsir A., Valea F., French D. L. (1995). Correlation of the in situ detection of polymerase chain reaction-amplified metalloproteinase complementary DNAs and their inhibitors with prognosis in cervical carcinoma.. Cancer Res.

[PDF_00728] Stetler-Stevenson W. G., Hewitt R., Corcoran M. (1996). Matrix metalloproteinases and tumor invasion: from correlation and causality to the clinic.. Semin Cancer Biol.

[PDF_00722] Stetler-Stevenson W. G., Krutzsch H. C., Liotta L. A. (1989). Tissue inhibitor of metalloproteinase (TIMP-2). A new member of the metalloproteinase inhibitor family.. J Biol Chem.

[PDF_00725] Stetler-Stevenson W. G., Liotta L. A., Kleiner D. E. (1993). Extracellular matrix 6: role of matrix metalloproteinases in tumor invasion and metastasis.. FASEB J.

[PDF_00731] Thorgeirsson U. P., Lindsay C. K., Cottam D. W., Gomez D. E. (1994). Tumor invasion, proteolysis, and angiogenesis.. J Neurooncol.

[PDF_00733] Welgus H. G., Jeffrey J. J., Eisen A. Z., Roswit W. T., Stricklin G. P. (1985). Human skin fibroblast collagenase: interaction with substrate and inhibitor.. Coll Relat Res.

[PDF_00736] Wilhelm S. M., Collier I. E., Marmer B. L., Eisen A. Z., Grant G. A., Goldberg G. I. (1989). SV40-transformed human lung fibroblasts secrete a 92-kDa type IV collagenase which is identical to that secreted by normal human macrophages.. J Biol Chem.

[PDF_00740] Yoshiji H., Gomez D. E., Thorgeirsson U. P. (1996). Enhanced RNA expression of tissue inhibitor of metalloproteinases-1 (TIMP-1) in human breast cancer.. Int J Cancer.

[PDF_00743] Zucker S., Lysik R. M., DiMassimo B. I., Zarrabi H. M., Moll U. M., Grimson R., Tickle S. P., Docherty A. J. (1995). Plasma assay of gelatinase B: tissue inhibitor of metalloproteinase complexes in cancer.. Cancer.

